# HER3, but Not HER4, Plays an Essential Role in the Clinicopathology and Prognosis of Gastric Cancer: A Meta-Analysis

**DOI:** 10.1371/journal.pone.0161219

**Published:** 2016-08-18

**Authors:** Guo-dong Cao, Ke Chen, Mao-ming Xiong, Bo Chen

**Affiliations:** 1 Anhui Medical University, Hefei, Anhui, 230022, China; 2 Department of General Surgery, The First Affiliated Hospital of Anhui Medical University, Hefei, Anhui, 230022, China; Istituto di Ricovero e Cura a Carattere Scientifico Centro di Riferimento Oncologico della Basilicata, ITALY

## Abstract

**Background and Aim:**

Human epidermal growth factor receptor (HER) family plays an important role in gastric cancer (GC), especially HER2. Too much attention has been paid to HER2; however, the functions of HER3 and HER4 overexpression in GC are always ignored. The clinicopathological and prognostic roles of HER3 and HER4 in GC are controversial. In this study, a systematic review and meta-analysis was conducted to evaluate the use of HER3 or HER4 as a predictor of clinicopathology and survival time in GC patients.

**Methods:**

Eligible studies were searched on PubMed, Ovid, Web of Science, and Cochrane databases through multiple search strategies. Data collection and statistical analysis were carried out by the Revman 5.3 software. The Newcastle-Ottawa scale was used to assess the quality of included studies.

**Results:**

A total of 448 studies about HER3 overexpression and GC, and 398 studies about HER4 overexpression and GC were searched. Of these, 5 eligible studies about HER3 including 1016 GC patients and 3 eligible studies about HER4 including 793 GC patients met the inclusion criteria. The results showed that HER3 and HER4 overexpression were significantly associated with depth of tumor invasion (OR = 0.44, 95%CI 0.29–0.67, *P* = 0.0002 and OR = 0.50, 95%CI 0.38–0.86, *P* = 0.007) and lymph node metastasis (OR = 0.40, 95%CI 0.20–0.77, *P* = 0.007 and OR = 0.57, 95%CI 0.38–0.86, *P* = 0.007), and HER3 overexpression reveals a tendency of later tumor node metastases (TNM) stage (OR = 0.50, 95%CI 0.22–1.15, *P* = 0.10) and predicts a worse survival time (RR = 0.71, 95%CI 0.61–0.84, *P*<0.00001), while HER4 overexpression had no correlation with TNM stage (OR = 0.60, 95%CI 0.20–1.78) and survival time (RR = 1.09, 95%CI 0.91–1.30).

**Conclusions:**

This meta-analysis indicated that HER3 plays an essential role in the clinicopathology and prognosis of GC. However, HER4 may not be an ideal prognostic factor for GC.

## Introduction

Gastric cancer (GC), one of the most common malignant tumors in the body, is the second cause of cancer-related deaths [1]. The early diagnosis rate of GC is low in Southeast Asia [2–3]. Most GC patients are at an advanced stage of cancer or distant metastasis, and even through the palliative surgical treatment, the 5–year overall survival(OS) is still optimistic, with the median OS being less than 1 year [4]. The prognosis of patients with advanced GC who received several new chemotherapeutic regimens is not ideal [5]. Therefore, it is necessary to find a new prognostic biomarker that could prolong the survival time of GC patients.

The human epidermal growth factor receptor (HER) family includes four members: epidermal growth factor receptor(EGFR)/HER1/ErbB1, HER2/ErbB2, HER3/ErbB3, and HER4/ErbB4. Compared with EGFR and HER2, the functions of HER3 in GC are always ignored. HER3 is distinct from the other three HER family members [6] in that it lacks intrinsic tyrosine kinase activity. Due to this feature of HER3, it cannot activate the intracellular signaling pathway by forming a homodimer [7]. Nevertheless, it usually heterodimerizes with other HER family members, especially HER2; the most active heterodimer is the HER2/HER3 dimer, which can activate the phosphoinositide 3-kinase/protein kinase B (PI3K/AKT) and the mitogen-activated protein kinase pathways in cancer [8–10]. As another member of HER family, HER4 overexpression in breast cancer is associated with significant worse survival in some studies [11–12], conversely, with better survival in other researches [13–14].

Several recent studies have reported that GC was closely linked with HER3 and HER4 expression [15–16]. Different researchers maintain different opinions on the associations of HER3 and HER4 with GC. Thus, several eligible studies were searched, and a systematic review was performed to evaluate the functions of HER3 and HER4 in GC.

## Methods

### Search strategy

The electronic databases from PubMed, Ovid, Web of Science, and Cochrane from January 1990 to January 2016 were searched. The search terms were as follows: ("HER3" or "ErbB3" or "HER4" or "ErbB4" or "Human epidermal growth factor receptor") and ("gastric" or "stomach" or "cardia" or "gastrointestinal") and ("adenocarcinoma" or "carcinoma" or "cancer" or "tumour" or "neoplasm" or "tumor"). The full texts of the studies were read to find whether the studies met the inclusion criteria.

### Inclusion and exclusion criteria

The inclusion criteria were as follows: (1) GC was identified, (2) HER3 or HER4 expression was evaluated by immunohistochemistry (IHC) assay, (3) information on clinicopathological parameters and OS was provided, (4)standards to assess the status of HER3 andHER4 was consistent in different studies, and (5) article was published in English or Chinese language. The exclusion criteria were as follows: (1) duplication, (2) reviews, (3) case reports, and (4) evaluation method was not IHC.

### Data extraction and quality assessment

According to the data selection criteria, all relevant data was extracted from each eligible study independently by two investigators (Guo-dong Cao, Ke Chen). During the process of data extraction, disagreements should be discussed with all research team members until a consistent opinion was reached. The following data were extracted:first author’s name, year of publication, total number of patients, the number of patients with HER3 or HER4 overexpression, clinicopathological parameters, and survival time. During the process of data extraction, disagreements were discussed with a third investigator (Mao-ming Xiong) until a consensus was reached. Two investigators (Guo-dong Cao and Ke Chen) assessed the quality of the included studies using the Newcastle–Ottawa scale [17]. No disagreement existed between the two researchers, and the quality of the included studies was re-evaluated by all authors, the scores of each study were recorded until reach consistent.

### Statistical analysis

The Revman 5.3 and STATA 11.0 software (Review Manager Version 5.3; The Nordic Cochrane Centre, Copenhagen, Denmark) were used for data analysis. Odds ratios and 95% confidence intervals (CIs) were used to estimate the association of HER3 and HER4 overexpression with clinicopathological parameters of GC patients. Risk ratiosand 95% CIs were used in this meta-analysis to evaluate the association of the status of HER3 and HER4 with OS. *I*^2^ value, which indicated the percentage of total variation across studies, was used to assess statistical heterogeneity. Heterogeneity among studies was assessed using the χ^2^ test (results were defined heterogeneous for *P*<0.10). The ORs were pooled using the random-effects model (DerSimonian–Laird method) [18] when statistical heterogeneity was found (*I*^2^>50% or *P*<0.10). Otherwise, the fixed-effects model (Mantel–Haenszel method) was considered [19]. Inconsideration of potential publication bias, Begg'srank correlation method and Egger's weighted regression method were conducted using STATA 11.0 software (*P*<0.05 indicates statistically significant publication bias).

## Results

### Study characteristics

**Fig 1:**
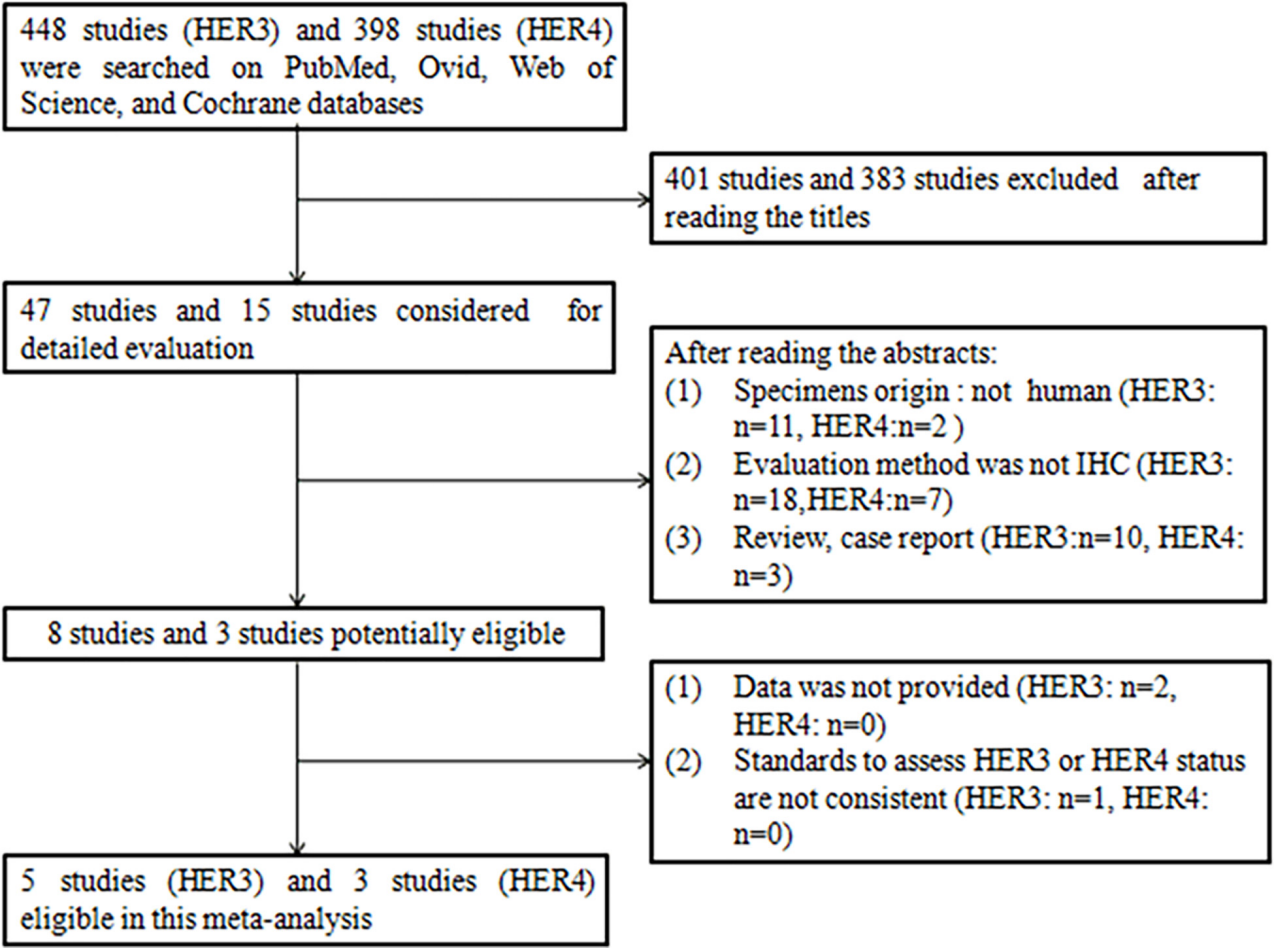
Flow diagram of study selection procedure.

A total of 448 studies about HER3 overexpression and GC, and 398 studies about HER4 overexpression and GC were searched. All 448 studies were published between August 1991 and January 2016. Of these, 47 studies and 15 studies, respectively, were potentially eligible after reading the title. After reviewing the abstracts and full texts based on the inclusion and exclusion criteria, five studies [20–24] and three studies [22,24,25], respectively, were finally chosen (Fig 1). The characteristics of these eligible publications are reported in Table 1. In Table 1, a total of five eligible studies about HER3 expression comprising 1,016 GC patients, ranging from 102 to 498 patients in different studies, were included. And a total of three studies about HER4 expression comprising 793 GC patients, ranging from 110 to 498 patients in different studies, were included. The clinical parameters such as depth of invasion, lymph node metastasis, TNM stage and Lauren’s type, are given in this table, the quality of the included studies was assessed according to the Newcastle–Ottawa scale is also performed in the table. The samples were analyzed using IHC, which was performed in all studies. The standards of assessing the status of HER3 and HER4 were almost consistent. The rates of HER3- and HER4-positive expression in patients with GC were 35.5% and 36.7%, respectively. The quality scores of the relevant studies assessed by Newcastle-Ottawa scale ranged from 5 to 8 stars. In the five studies about HER3 expression, three articles scored 5 stars, two articles scored 8 stars. In the three studies about HER4 expression, one articles scored 5 stars, two articles scored 8 stars. If articles achieved as core of six or more, they were considered of high quality.

**Table 1:** Clinicopathological parameters and quality scores of studies comparing HER3/HER4 positive GC with HER3/HER4 negative GC in Asian population.

HER family members	Study	Year	Number of Patients	Sex	Age	Tumor location	Depth of invasion	LN metastasis	Distant metastasis	TNM stage	Recurrence	Lauren’s type	Vascular invasion	Quality score^a^
				(male/female)		(cardia/body+antrum)	(T1+T2/T3+T4)	(N0/N1+N2+N3)	(M0/M1)	(I+II/III+IV)	(negative/positive)	(intestinal/diffuse)	(negative/positive)	
HER3	Hayashi	2008	134(79 *vs*.55)	51/28*vs*.43/12	67*vs*.64	NA	NA	21/58*vs*.41/14	64/15*vs*.53/2	NA	41/25*vs*.51/3	27/52*vs*.22/33	NA	8
	Zhang	2009	102(14 *vs*.88)	NA	NA	NA	NA	NA	NA	3/11*vs*.49/39	NA	3/11*vs*.57/31	NA	5
	Wu	2014	161(90 *vs*.71)	73/17*vs*.51/20	NA	43/47*vs*.28/43	19/71*vs*.30/41	31/59*vs*.37/34	81/9*vs*.68/3	NA	NA	18/72*vs*.18/53	37/53*vs*.41/30	5
	Tang	2015	121(75 *vs*.46)	52/23*vs*.33/13	NA	NA	8/67*vs*.3/43	17/58*vs*.14/32	70/5*vs*.42/4	23/52*vs*.13/33	NA	20/55*vs*.4/40	NA	5
	He	2015	498(103*vs*.395)	70/33*vs*.280/115	60*vs*.58	52/51*vs*.247/148	13/90*vs*.109/286	23/80*vs*.134/261	86/17*vs*.322/73	26/77*vs*.180/215	NA	88/15*vs*.196/199	NA	8
HER4	Hayashi	2008	134(115*vs*.19)	80/35*vs*.14/5	66*vs*.66	NA	NA	51/64*vs*.11/8	103/12*vs*.14/5	57/58*vs*.9/10	81/24*vs*.11/4	46/69*vs*.3/16	NA	8
	Li	2013	161(110*vs*.51)	82/28*vs*.42/9	NA	52/58*vs*.19/32	28/82*vs*.21/30	68/42*vs*.25/26	105/5*vs*.44/7	18/92*vs*.18/33	NA	23/87*vs*.13/38	49/61*vs*.29/22	5
	He	2015	498(66*vs*.432)	45/21*vs*.305/127	60*vs*.59	39/27*vs*.260/172	10/56*vs*.112/320	14/52*vs*.143/289	54/12*vs*.354/78	NA	NA	56/10*vs*.228/204	NA	8

Abbreviations: HER: human epidermal growth factor receptor; GC: gastric cancer; vs.: versus; NA: not available; TNM: depth of invasion (T), lymph nodes metastasis (N), and presence of distant metastasis (M).

T1-T4, N0-N3, M0-M1and TNM stages are based on tumor-node-metastasis classification advocated by International Union against Cancer

Quality score^a^: use the Newcastle-Ottawa scale (stars)

### Correlation of HER3 and HER4 with clinicopathological parameters of GC patients

**Fig 2:**
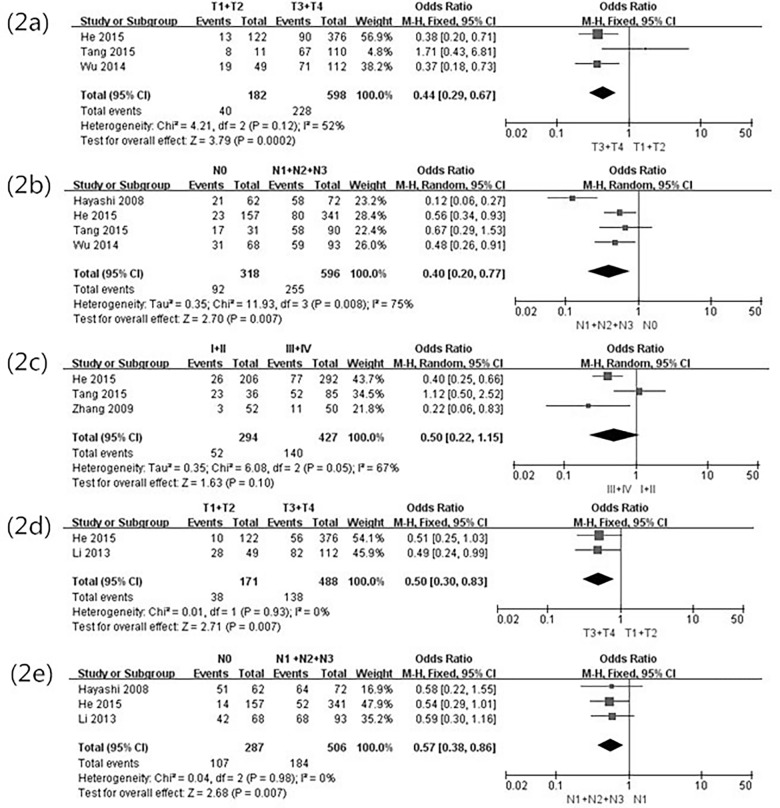
Forest plot of odds ratio for the association of HER3, HER4 over-expression and clinicopathological parameters. (2a) Association between HER3 over-expression and depth of invasion. (2b) Association between HER3 over-expression and lymph node metastasis. (2c) Association between HER3 over-expression and TNM stage. (2d) Association between HER4 over-expression and depth of invasion. (2e) Association between HER4 over-expression and lymph node metastasis.

The positive rates of HER3 in the five studies ranged from 13.7% to 62.0%, and the rate of HER3-positive expression in total GC patients was 35.5% (361/1,016). The correlation of HER3 expression with clinicopathological parameters is shown in Table 2. When the data were pooled, a significant association was found between HER3 over- expression and clinical variables. The combined data showed that the HER3-positive expression was tightly related to the depth of tumor invasion (OR = 0.44, 95%CI 0.29–0.67, *P* = 0.0002) (Fig 2A), lymph node metastasis (OR = 0.40, 95%CI 0.20–0.77, *P* = 0.007) (Fig 2B), recurrence (OR = 0.10, 95%CI 0.03–0.34, *P*<0.0001), and vascular invasion(OR = 0.51, 95%CI 0.27–0.96, *P* = 0.026). It is worth noting that HER3 overexpression probably means a tendency of later TNM stage (OR = 0.50, 95%CI 0.22–1.15, *P* = 0.10) (Fig 2C). HER3 overexpression was not related to gender, tumor location, distant metastasis, or Lauren’s type.

**Table 2:** Association between clinicopathological parameters and HER3/HER4 over-expression in GC.

HER family members	Parameters	Number of studies	Number of patients	Heterogeneity		Model	OR(95%CI)	*P* value
				*I*^2^(%)	*P* value			
HER3	Sex(male/female)	4	914	37	0.19	FE	0.90(0.66,1.24)	0.53
	Tumor location(cardia/body+autrum)	2	659	78	0.03	RE	0.90(0.40,2.02)	0.79
	Depth of invasion(T1+T2/T3+T4)	3	780	52	0.12	FE	0.44(0.29,0.67)	**0.0002**
	LN metastasis(N0/N1+N2+N3)	4	914	75	0.008	RE	0.40(0.20,0.77)	**0.007**
	Distant metastasis(M0/M1)	4	914	60	0.06	RE	0.64(0.26,1.58)	0.33
	TNM stage (I+II/III+IV)	3	721	67	0.05	RE	0.50(0.22,1.15)	0.1
	Recurrence (negative/positive)	1	120	–	–	–	0.10(0.03,0.34)	**<0.0001**
	Lauren’s type (intestinal/diffuse)	5	1014	90	<0.00001	RE	1.19(0.36,3.95)	0.78
	Vascular invasion(negative/positive)	1	161	–	–	–	0.51(0.27,0.96)	**0.026**
HER4	Sex(male/female)	3	793	0	0/79	FE	0.80(0.52,1.22	0.3
	Tumor location(cardia/body+autrum)	2	659	8	0.3	FE	1.14(0.75,1.72)	0.55
	Depth of invasion(T1+T2/T3+T4)	2	659	0	0.93	FE	0.50(0.30,0.83)	**0.007**
	LN metastasis(N0/N1+N2+N3)	3	793	0	0.98	FE	0.57(0.38,0.86)	**0.007**
	Distant metastasis(M0/M1)	3	793	56	0.1	RE	1.92(0.80,4.58)	0.14
	TNM stage (I/II+III+IV)	2	295	68	0.08	RE	0.60(0.20,1.78)	0.36
	Recurrence (negative/positive)	1	120	–	–	–	1.23(0.36,4.21)	0.749
	Lauren’s type (intestinal/diffuse)	3	793	84	0.002	RE	2.35(0.64,8.61)	0.2
	Vascular invasion(negative/positive)	1	161	–	–	–	0.61(0.31,1.19)	0.099

Abbreviations: OR: odds ratio; CI: confidence interval; FE: fixed-effect model; RE: random-effect model; LN metastasis: lymph node metastasis

The positive rates of HER4 in the three studies range from 13.3% to 85.8%, and the rate of HER4-positive expression in all patients with GC was 36.7% (291/793). The correlation of HER4 expression with clinicopathological parameters is shown in Table 2. HER4 overexpre- ssion was associated with the depth of tumor invasion (OR = 0.50, 95%CI 0.30–0.83, *P* = 0.0007) (Fig 2D) and lymph node metastasis (OR = 0.57, 95%CI 0.38–0.86, *P* = 0.0007) (Fig 2E). HER4 overexpression was not related to gender, tumor location, distant metastasis, TNM stage, recurrence, Lauren’s type, or vascular invasion.

### Correlation of HER3 and HER4 overexpression with OS

**Fig 3:**
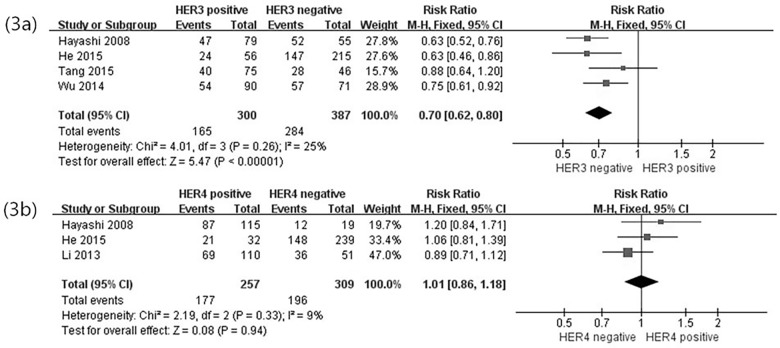
Forest plot of risk ratio for the association of HER3, HER4 over-expression and OS. (3a) Association between HER3 over-expression and 3-year OS. (3b) Association between HER4 over-expression and 3-year OS.

Kaplan–Meier survival curves were used to assess the effect of HER3 expression on the OS in the five studies. Among these studies, only one study found no significant correlation between HER3 overexpression and OS, whereas the other four studies exhibited a significant association between HER3 overexpression and survival time of the patients. In this study, the correlation of HER3 overexpression with 1-, 3-, and 5-year OS was determined (Table 3). The risk ratio(RR = 0.70, 95%CI 0.62–0.80, *P*<0.00001) estimated by a fixed-effects model demonstrated a lower rate of 3-year OS in the HER3-positive GC patients than in the HER3-negative GC patients (Fig 3A). No statistical heterogeneity (*P =* 0.48, *I*^2^ = 0%) was found in these five studies.

**Table 3:** Association between HER3/HER4 over-expression and overall survival (OS) in GC.

HER family members	OS	Number of studies	Number of patients	Heterogeneity		Model	OR(95%CI)	*P* value
				*I*^2^(%)	*P* value			
HER3	1-year OS	5	789	0	0.78	FE	0.89(0.83,0.96)	**0.002**
	3-year OS	4	687	25	0.26	FE	0.70(0.62,0.80)	**<0.00001**
	5-year OS	3	566	44	0.17	FE	0.71(0.61,0.84)	**<0.00001**
HER4	1-year OS	3	566	28	0.25	FE	1.03(0.93,1.14)	0.55
	3-year OS	3	566	9	0.33	FE	1.01(0.86,1.18)	0.94
	5-year OS	3	566	0	0.52	FE	1.09(0.91,1.30)	0.37

Abbreviations: OS: overall survival; RR: risk ratio; CI: confidence interval; FE: fixed-effect model; RE: random-effect model

The three included studies about HER4 expression provided Kaplan–Meier survival curves; the survival time was obtained from these studies and then the data was pooled. No association was found between HER4 overexpression and OS (Table 3; Fig 3B). In summary, HER3, but not HER4, should be regarded as a valuable prognostic factor in GC.

### Publication bias analysis

**Fig 4:**
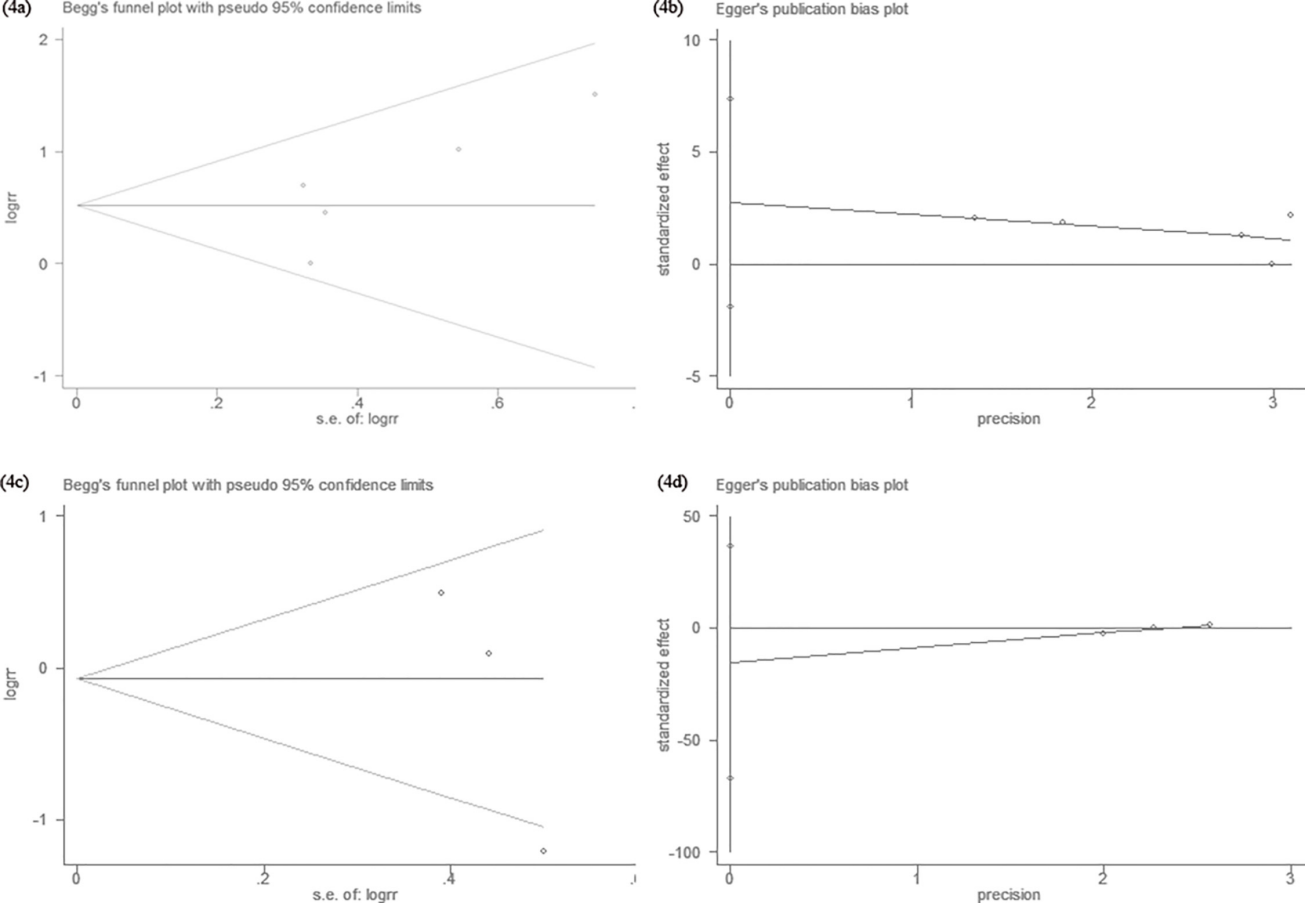
Begg’s funnel plot and Egger’s funnel plot for possible publication bias test of this study. There was no publication bias and the results are credible. (4a) Begg’s test of HER3 overexpression and OS (*P* = 0.14). (4b) Egger’s test of HER3 overexpression and OS (*P* = 0.15). (4c) Begg’s test of HER4 overexpression and OS (*P* = 0.12). (4d) Egger’s test of HER4 overexpression and OS (*P* = 0.17).

Begg's rank correlation method and Egger's weighted regression method were used to statistically assess publication bias. As shown in Fig 4A and 4B, neither Begg’s (HER3: *P* = 0.14, HER4: *P* = 0.12) nor Egger’s (HER3: *P* = 0.15, HER4: *P* = 0.17) test provided any clear evidence of publication bias. These results indicate that there was no publication bias in the current study, and that the results reported in this meta-analysis are credible.

## Discussion

The main factors that impact the clinicopathological parameters and prognosis of GC patients are not entirely clear. The HER family members play important roles in GC, and significantly affect the prognosis of GC patients. In these four HER family members, previous meta-analyses have investigated the prognostic value of HER2 [26] and HER3 [27] in tumors. It should be noted that, no paper has studied the association between HER3/HER4 high expression and the clinical/prognostic value of GC patients. In the meta-analysis, a significant association was found between HER3 overexpression and depth of tumor invasion, lymph node metastasis, recurrence, and vascular invasion. Moreover, HER3 overexpression always indicates a shortened OS. However, no association was found between HER4 overexpression and clinical variables or OS.

As is known, surgery still remains the primary strategy in the cancer treatment, whereas not all patients could benefit from a radical resection. Adjuvant systemic chemotherapies have been developed to improve this poor perspective [28]. The survival time of GC patients whose tumor cannot be removed has been prolonged by new regimens including taxanes, irinotecan, and novel fluoropyrimidines [5,29]. However, even after the palliative surgical treatment and chemotherapy, the median OS was less than 1 year [4–5]. Researchers have great interest in identifying prognostic factors in patients with GC. HER2 is an important member of the HER family. Its gene amplification and protein overexpression in GC were first reported in 1986 [30], and then more and more attention was paid on the HER family. Four HER family receptors share a common structure of an extracellular ligand-binding domain, transmembrane domain, and an intracellar tyrosine kinase domain [7,31–32]. Distinct from other HER family members, HER3 lacks intrinsic tyrosine kinase activity [6,8]. As a result, HER3 was always neglected by researchers. Overexpression of HER3 was found in several types of cancers, including bladder, prostate, and breast cancers [33–34], and its positive expression was significantly correlated with the decreased survival time [35–36]. A similar situation happened with HER4. This study investigated whether associations existed among the status of HER3 and HER4 and clinicopathological parameters and the survival time of GC patients by a meta-analysis.

The TNM stage—depth of invasion (T), lymph nodes metastasis (N), and presence of distant metastasis (M)—was considered as the most important prognostic factor for GC [37]. In the present meta-analysis, HER3 overexpression was significantly related to the depth of tumor invasion, lymph node metastasis, recurrence, and vascular invasion. In laryngeal and esophageal cancers, HER3 overexpression is significantly associated with involved lymph nodes [38–39]. Lymph node metastasis was considered as an important prognostic factor, which increases the risk of recurrence after surgery. Seo et al reported that HER3 was tightly associated with the TNM stage in colorectal cancer (CRC) [40]. This study found that HER3 overexpression might lead to a later TNM stage (*P* = 0.10) in GC. It is credible that HER3 plays an essential role in the decreased survival, and is related to clinicopathology. In contrast to HER2, not all clinicopathological variables are significantly related with HER3 overexpression. HER3 overexpression was not correlated with gender, tumor location, distant metastasis, or Lauren’s type. In CRC, HER3 also has no correlation with gender and tumor location, and these parameters were not regarded as the main promising factors in cancer. In lots of researches about GC, Lauren’s type was regarded as a prognostic factor; it is meaningful to survival time. Begnami et al [41] have reported that HER3 overexpression was significantly associated with the intestinal subtype of GC; however, other researchers [15,42] maintained an opposite opinion that no significant relationship exists between HER3 expression and Lauren’s classification. So HER3 probably plays a limited function in GC. Consistent with other studies, HER3 overexpression is significantly associated with the survival time of the patients; HER3-positive GC patients have a shorter survival time.

The trastuzumab for gastric cancer (ToGA) trial was a clinical phase 3 trial that compared the HER2 monoclonal antibody (trastuzumab) plus chemotherapy with chemotherapy alone for treating HER2-positive advanced gastric or gastroesophageal junction cancer. Although the ToGA trial showed that HER2 monoclonal antibody (trastuzumab) can improve the survival time of advanced GC, only 12.8% of HER2-positive GC patients could benefit from trastuzumab. The result indicated that HER2-positive GC patients exhibit resistance to trastuzumab [43]. HER3 overexpression may significantly be associated with trastuzumab resistance; acquisition of trastuzumab resistance was associated with a higher expression of HER3[44]. Li et al [45] also concluded that anti-HER3 monoclonal antibody can reduce trastuzumab resistance in gynecological cancers.

The limited function of HER3 and trastuzumab resistance in GC can be attributed to the facts that HER2 has no ligand and HER3’s intrinsic tyrosine kinase domain is defective, HER3 usually heterodimerizes with HER2, and the HER2/HER3 heterodimer is likely to be the most effective complex of all the heterodimers [46–47]. HER3 is a favored dimerization partner that can sustain the activation of PI3K/AKT signaling pathway [48–49]. However, because of its own deficiency, if only HER3-positive expression alone exists without the overexpression of other HER family members, HER3 may just play a limited function and affect the clinicopathology and survival time in GC patients.

Further, the studies about HER4 overexpression in cancers were relatively insufficient. It is also controversial whether HER4 overexpression is related to clinicopathology and OS. Kountourakis et al [50] reported that HER4 was not associated with the prognosis of patients with colorectal cancer. Nielsen et al [51] found the mRNA expression of HER4 to be downregulated in the tumor tissue compared with the matched normal tissue. A similar conclusion was obtained in the present meta-analysis that HER4-positive expression is meaningless in GC patients. HER4 overexpression was related to the depth of tumor invasion and lymph node metastasis, whereas it was not related to gender, tumor location, distant metastasis, TNM stage, recurrence, Lauren’s type, or vascular invasion. No association was found between HER4 overexpression and OS in this study.

Similar to other system reviews, this study also had three several limitations. First, several relevant articles were searched through a comprehensive literature search strategy, with the article language limited to English and Chinese. Due to this, some eligible non-English and non-Chinese publications may have been excluded. Second, not all studies were rated as high quality. Third, the number of articles about HER3 and HER4 overexpression in GC is still insufficient, with some clinicopathological parameters mentioned in only one study, so the results may have exhibited biases. Forth, IHC assessments of HER3 and HER4 were still discordant. Fifth, some findings have significant heterogeneity (*I*^2^>50% or *P*<0.10).

In spite of the limitations in this study, several advantages of this meta-analysis should be acknowledged: (1) This study is the first available system review on the association between HER3 overexpression and clinicopathological parameters in GC. (2) Whether HER4 overexpression was correlated with clinicopathological parameters and OS in GC was investigated. (3) Some findings have zero heterogeneity, the results were obviously credible. For example, the 1-year OS has zero heterogeneity (*I*^2^ = 0% or *P* = 0.78), the result was accurate in the opinion of the authors. (4) To minimize possible biases, a comprehensive literature search strategy was made, by searching relevant studies meticulously and designing a detailed protocol for data collection and analysis.

## Conclusion

In summary,this meta-analysis revealed that HER3 is significantly associated with clinicopathology and OS, and plays an essential function in GC patients. Thus, HER3 could become a new promising chemotherapeutic biomarker. However, HER4 has no correlation with the prognosis of this disease.

## Supporting Information

S1 PRISMA Checklist PRISMA 2009 checklist [Update]. (DOC)
